# Associations between Genetic Variants in the Vitamin D Metabolism Pathway and Severity of COVID-19 among UAE Residents

**DOI:** 10.3390/nu13113680

**Published:** 2021-10-20

**Authors:** Fatme Al-Anouti, Mira Mousa, Spyridon N. Karras, William B. Grant, Zainab Alhalwachi, Laila Abdel-Wareth, Maimunah Uddin, Nawal Alkaabi, Guan K. Tay, Bassam Mahboub, Habiba AlSafar

**Affiliations:** 1 Department of Health Sciences, College of Natural and Health Sciences, Zayed University, Abu Dhabi, United Arab Emirates; Fatme.AlAnouti@zu.ac.ae; 2 Nuffield Department of Women’s and Reproductive Health, Oxford University, Oxford OX1 4BH, UK; mira.mousa@stx.ox.ac.uk; 3 Center for Biotechnology, Khalifa University of Science and Technology, Abu Dhabi, United Arab Emirates; zainabhhalwachi@gmail.com (Z.A.); guan.tay@uwa.edu.au (G.K.T.); 4 National Scholarship Foundation, 55535 Thessaloniki, Greece; karraspiros@yahoo.gr; 5 Sunlight, Nutrition and Health Research Center, San Francisco, CA 94164-1603, USA; williamgrant08@comcast.net; 6 National Reference Laboratory, Abu Dhabi, United Arab Emirates; WarethL@ClevelandClinicAbuDhabi.ae; 7 Pathology and Laboratory Medicine Institute, Cleveland Clinic Abu Dhabi, Abu Dhabi, United Arab Emirates; 8 Department of Pediatric Infectious Disease, Sheikh Khalifa Medical City, Abu Dhabi, United Arab Emirates; muddin@seha.ae (M.U.); nalkaabi@seha.ae (N.A.); 9 Psychiatry, Medical School, University of Western Australia, Perth, WA 6009, Australia; 10 School of Medical and Health Sciences, Edith Cowan University, Joondalup, WA 6027, Australia; 11 Dubai Health Authority, Rashid Hospital, Dubai, United Arab Emirates; bhmahboub@dha.gov.ae; 12 Department of Biomedical Engineering, College of Engineering, Khalifa University of Science and Technology, Abu Dhabi, United Arab Emirates

**Keywords:** vitamin D, metabolism, polymorphism, COVID-19, UAE, genotypes

## Abstract

Vitamin D has many effects on cells in the immune system. Many studies have linked low vitamin D status with severity of COVID-19. Genetic variants involved in vitamin D metabolism have been implicated as potential risk factors for severe COVID-19 outcomes. This study investigated how genetic variations in humans affected the clinical presentation of COVID-19. In total, 646 patients with SARS-CoV-2 infection were divided into two groups: noncritical COVID-19 (*n* = 453; 70.12%) and a critical group (*n* = 193; 29.87%). Genotype data on the GC, NADSYN1, VDR, and CYP2R1 genes along with data on serum 25-hydroxyvitamin D levels were compiled in patients admitted to a major hospital in the United Arab Emirates between April 2020 and January 2021. We identified 12 single-nucleotide polymorphisms associated with the critical COVID-19 condition: rs59241277, rs113574864, rs182901986, rs60349934, and rs113876500; rs4944076, rs4944997, rs4944998, rs4944979, and rs10898210; and rs11574018 and rs11574024. We report significant associations between genetic determinants of vitamin D metabolism and COVID-19 severity in the UAE population. Further research needed to clarify the mechanism of action against viral infection in vitamin D deficiency. These variants could be used with vaccination to manage the spread of SARS-CoV-2 and could be particularly valuable in populations in which vitamin D deficiency is common.

## 1. Introduction

In late 2019, the first cases of COVID-19, caused by the severe acute respiratory syndrome coronavirus 2 (SARS-CoV-2), were reported in Wuhan, China [1]. Phenotypic heterogeneity has been observed in COVID-19 cases, with symptoms characterized as asymptomatic, mild, moderate, and severe, with evidence that the hyperinflammatory responses induced by SARS-CoV-2 are the major causes of disease severity and death [2]. In addition, several studies have shown that individuals with comorbidities, such as obesity, hypertension, type 2 diabetes, and cardiovascular diseases, are more susceptible to developing severe COVID-19 symptoms owing to an imbalance in the immune response and uncontrolled release of proinflammatory cytokines [3]. While worldwide vaccination efforts are still underway with unequal and mismatched rates among countries, efforts continue to be directed toward treatments, which aim to reduce the number of severe cases and deaths.

One strategy to reduce severe COVID-19 symptoms relies on high-dose biological factors, vitamins, and trace elements, such as vitamin D, zinc, and selenium [4]. Vitamin D has been identified as an important molecule in attenuating the SARS-CoV-2 viral infection through binding to the vitamin D receptor (*VDR*) to control the immune response [5,6]. Moreover, vitamin D had been identified by novel genomics-guided tracing research as a promising candidate molecule through orchestrating the expression of immune-related genes with potential to reduce SARS-CoV-2 viral infection through binding to the VDR [7]. Specific variants in genes involved in vitamin D metabolism have been identified as potential risk factors for severe COVID-19 outcomes [8], but research in this area remains limited.

We recently showed that severe vitamin D deficiency strongly correlated with COVID-19 severity and death among patients in the UAE [9]. Furthermore, genetic polymorphisms in the *VDR* gene affect the susceptibility to certain chronic diseases, such as diabetes and obesity [10]. Therefore, genetic variants in vitamin D metabolism and *VDR* genes could be important factors in determining COVID-19 disease severity and progression. This study aimed to examine the association of polymorphisms in *VDR* and other genes involved in vitamin D metabolism with COVID-19 disease outcome among the UAE population.

## 2. Materials and Methods

### 2.1. Participants and Collecting Samples

A total of 646 participants who tested positive for SARS-CoV-2 by real-time PCR via nasopharyngeal swabs were recruited from the Sheikh Khalifa Medical City, quarantine area in Abu Dhabi, and Rashid Hospital, in Dubai, between April 2020 and January 2021. This study was approved by the Abu Dhabi Health COVID-19 Research Ethics Committee (DOH/DQD/2020/538), the Dubai Scientific Research Ethics Committee (DSREC-04/2020_09), and the SEHA Research Ethics Committee (SEHA-IRB-005). Details about participant recruitment and data collection were outlined previously [9].

### 2.2. Collecting Demographic Data

Clinical and demographic data were collected from participants and medical staff at the hospital and quarantine area. Participants were divided into two groups: noncritical patients (*n* = 453) and critical patients (*n* = 193). Noncritical illness was defined as nonhospitalized patients with asymptomatic to mild symptoms. Critical illness was defined as patients who have SpO_2_ <94% in room air at sea level, a ratio of arterial partial pressure of oxygen to fraction of inspired oxygen (PaO_2_/FiO_2_) <300 mmHg, respiratory frequency >30 breaths/min, or lung infiltrates >50% that require hospital admission because of use of mechanical ventilation, supplemental oxygen therapy, progression to respiratory failure, and severe complications, such as septic shock or multiorgan failure. Among participants who reported a comorbidity, critical COVID-19 patients were more likely to have had a previous diagnosis of cardiovascular disease (arrhythmias, congenital heart disease, coronary artery disease, deep vein thrombosis, myocardial infarction, cardiomyopathy; *p* < 0.001) or chronic lung disease (asthma, pneumonia, bronchitis, pneumothorax).

### 2.3. DNA Extracting and Genotyping

A total of 2 mL of blood was collected by a qualified nurse in an EDTA tube. Samples were transferred to the Khalifa University Center for Biotechnology’s laboratory for DNA extraction and genotyping. DNA was extracted according to the manufacturer’s instructions. DNA was quantified using a DS-11 Series spectrophotometer/fluorometer (DeNovix, Wilmington, Delaware, USA). Genotyping was performed using the Infinium Global Screening Array (Illumina, San Diego, CA, USA) according to the manufacturer’s protocol. The Infinium array contained 654,027 genetic markers. Genotypes were prephased and imputed using the Phase 3 1000 Genomes Projects panel, carried out with BEAGLE, using standard protocols and recommended settings. Post-imputation quality controls were conducted, and 9,175,654 variants passed filters. The locations of the following single-nucleotide polymorphisms (SNPs) were extracted: *GC* in chromosome 4, *DHCR7*/*NADSYN1* in chromosome 11, *VDR* in chromosome 12, and *CYP2R1* in chromosome 11.

### 2.4. Measuring Serum 25(OH)D Levels

A total of 2 mL of blood was collected by a qualified nurse in a gel tube to measure levels of total 25-hydroxyvitamin D [25(OH)D] with automated electrochemiluminescence (Elecsys 2010; Roche Diagnostics, GmbH, Mannheim, Germany). The detection limit of serum 25(OH)D was 4 ng/mL. The intra-assay coefficient of variation was 5%, and the interassay coefficient was 7.5%.

### 2.5. Statistical Analysis

Statistical analysis was conducted as a case–control panel, with controls characterized as noncritical symptoms and cases characterized as critical symptoms, using PLINK (version 1.9) and SPSS (version 16.0; SPSS, Chicago, Illinois, USA). Cross-tabulation and chi-square test were used to evaluate the association between clinical severity of disease with symptomatology and risk factors. Genomewide association study (GWAS) screening was performed with the χ^2^ statistic. To control for population stratification, the first 10 eigenvectors were used in later adjustment analyses. The adjusted analysis corrected for age, sex, and population stratification. To assess the association of a given SNP with COVID-19 severity, the allelic frequencies were compared by means of a χ^2^ statistic, which yielded an individual *p*-value for each combination of SNP and genetic model. The *p*-values were not adjusted for multiple testing because of the explorative nature of the original GWAS. Haplotype analysis was performed to estimate the genetic contribution of haplotypes to COVID-19 severity, which can be more informative and powerful than the association of individual variants.

## 3. Results

Table 1 summarizes demographic data of the 646 participants, divided as described above. Critical COVID-19 patients were older (*p* < 0.001), with higher body mass index (BMI; kilograms per square meter of body surface area; *p* = 0.001), of Middle Eastern or Asian ethnicities (*p* = 0.004), and suffering from one or more comorbidities (*p* < 0.001). Male patients were not at higher risk of developing severe/critical disease than female patients (*p* = 0.22). Among participants who reported a comorbidity, critical COVID-19 patients were more likely to have had a previous diagnosis of cardiovascular disease (arrhythmias, congenital heart disease, coronary artery disease, deep vein thrombosis, myocardial infarction, cardiomyopathy; *p* < 0.001) or chronic lung disease (asthma, pneumonia, bronchitis, pneumothorax).

To investigate the association of 25(OH)D with risk of critical COVID-19 disease, serum 25(OH)D levels were measured in some participants (Table 2). After adjustment for age, sex, nationality, and BMI, critical COVID-19 patients were more likely to be severely vitamin D deficient (OR = 4.37 [95% CI, 2.06–9.29]; *p* < 0.001) than noncritical COVID-19 patients.

To further investigate genetic effects between COVID-19 phenotype and vitamin D, all SNPs (total of 578) located in the *GC* gene (chromosome 4, 72607410–72671237; 168 SNPs), *DHCR7*/*NADSYN1* (chromosome 11, 71164249–71212862; 230 SNPs), *VDR* (chromosome 12, 48235320–48298777; 154 SNPs), and *CYP2R1* (chromosome 11, 14898986–14913777; 9 SNPs) were extracted. Those SNPs are involved in synthesis, transport, and metabolism of vitamin D and the VDR. The COVID-19 phenotype was dichotomously classified into the critical group (*n* = 453) and the noncritical group (*n* = 193). Imputation in this multiancestry cohort was performed to increase power. Thus, SNPs included in the dataset were imputed SNPs and tag SNPs. Table 3 shows the top five SNPs significant for each gene, and the remaining SNPs are listed in Supplementary Table S1. Supplementary Table S2 includes the genotype data and genetic model, whereas Supplementary Table S3 includes the haplotype association model.

Specific SNPs located in the genes *GC*, *DHCR7*/*NADSYN1*, and *VDR* were associated with the critical COVID-19 condition among patients. However, no SNPs examined in gene *CYP2R1* were associated with risk of critical COVID-19 condition. For gene *GC*, the AA genotype in SNP rs59241277 (*p* = 0.005), CC genotype in SNP rs113574864 (*p* = 0.005), GG genotype in SNP rs182901986 (*p* = 0.01), TT genotype in SNP rs60349934 (*p* = 0.01), and GG genotype in SNP rs113876500 (*p* = 0.02) were associated with the critical COVID-19 condition. For gene *DHCR7*/*NADSYN1*, AA genotype in SNP rs4944076 (*p* = 0.008), GG genotype in SNP rs4944997 (*p* = 0.02), GG genotype in SNP rs4944998 (*p* = 0.02), GG genotype in SNP rs4944979 (*p* = 0.02), and AA genotype in SNP rs10898210 (*p* = 0.009) were similarly associated with severity, whereas for gene *VDR*, TT genotype in SNP rs11574018 (*p* = 0.04) and GG genotype in SNP rs11574024 (*p* = 0.04) were significant.

As shown in Supplementary Table S1, 20 blocks are incorporated within the four genomic regions: *GC*, *DHCR7*/*NADSYN1*, *CYP2R1*, and *VDR.* The following haplotype windows (Supplementary Table S3) were associated with the critical COVID-19 condition among patients: *VDR* gene (block 7 [*p* = 0.01], block 10 [*p* = 0.04]), *DHCR7/NADSYN1* gene (block 11 [*p* = 0.03]), and *GC* gene (block 15 [*p* = 0.003; *p* = 0.0004], block 16 [*p* = 0.003; *p* = 0.003], block 17 [*p* = 0.003], block 18 [*p* = 0.003; *p* = 0.03], block 19 [*p* = 0.02], block 20 [*p* = 0.004; *p* = 0.002]).

## 4. Discussion

To the best of our knowledge, this study is the first to examine the contribution of genetic variants of vitamin D metabolism to COVID-19 disease severity. Specifically, 25(OH)D levels, which reflect vitamin D status in the body, were measured in a cohort of patients who tested positive for SARS-CoV-2 in the UAE. With 63% of critical patients having deficient or severely deficient vitamin D levels, the plausible association with severity of viral infections was examined. These findings also accord with findings associating lower vitamin D levels (<12 ng/mL) with increased risk of critical COVID-19 condition [9]. Specific variants in genes involved in vitamin D metabolism have been proposed as potential risk factors for severe COVID-19 outcomes [11], but specific research in this context remain limited. In this study, we analyzed several variants in the loci near protein-coding genes involved in the vitamin D pathway in a cohort of SARS-CoV-2-positive patients from the UAE population to investigate the possible association with COVID-19 disease severity. The data presented here indicated a significant correlation between specific variants of the GC, VDR, and DHCR7/NADSYN1 genes and susceptibility to severe COVID-19 infection.

The results showed that being older, having higher BMI, and the presence of one or more comorbidities were important risk factors for developing a critical COVID-19 condition among SARS-CoV-2-positive patients. These findings are consistent with previously reported data regarding COVID-19 susceptibility and risk factors from several populations [12,13]. Moreover, severe vitamin D deficiency was an independent risk factor for the critical condition, in accordance with previous reports [14,15,16].

Genotype data were analyzed in relation to patients’ vitamin D levels, revealing the involvement of vitamin D homeostasis and its metabolic pathway in influencing susceptibility to severe COVID-19 disease. These genotypic differences in COVID-19 disease outcome could be attributed to vitamin D’s role in host immunity against SARS-CoV-2 or other viral infections [17,18].

The results here also highlighted the genetic contribution of specific haplotypes for VDR, DHCR7/NADSYN1, and GC genes to COVID-19 critical condition, emphasizing the importance of genotypic variations in determining disease severity in populations. For example, the AA genotype in SNP rs59241277, CC genotype in SNP rs113574864, GG genotype in SNP rs182901986, TT genotype in SNP rs60349934, and GG genotype in SNP rs113876500 in gene GC were associated with critical COVID-19 condition. The AA genotype in SNP rs4944076, GG genotype in SNP rs4944997, GG genotype in SNP rs4944998, GG genotype in SNP rs4944979, and AA genotype in SNP rs10898210 in DHCR7/NADSYN1 gene were similarly associated with severity, whereas for gene VDR, the TT genotype in SNP rs11574018 and GG genotype in SNP rs11574024 were significant.

A recent study by Kotur and colleagues (2021) reported a significant association between specific genetic variants of DHCR7/NADSYN1 rs12785878, GC rs2282679, *CYP2R1* rs10741657, and *VDR* rs2228570 and severe clinical outcomes among Serbian adults but not among pediatric patients [8]. The findings in this study are consistent with results of their study, though in this study no significant correlation was evident between COVID-19 clinical conditions and *CYP2R1* genetic variants.

AlSafar and colleagues (2021) recently reported that severe vitamin D deficiency strongly correlated with COVID-19 severity and death among the UAE population [9]. Furthermore, several studies have shown that genetic polymorphisms in the VDR gene affect susceptibility to certain chronic diseases, such as diabetes and obesity [10]. Our group investigated the association of genetic variants within VDR and other genes important in vitamin D metabolism with type 2 diabetes among Emiratis and concluded that a strong association existed with risk of disease for specific variants [19,20]. In a similar context, we showed the implications of such genetic variants in susceptibility to obesity among young Arabs in the UAE [21]. Thus, exploring the relation of genetic variants involved in vitamin D metabolism and COVID-19 severity is worth considering and might offer population-based tools to identify individuals at greater risk of developing severe disease outcomes. Such strategies, which depend on functional polymorphism, could lead to implementing future protective measures in conjunction with vaccination [4,5,8].

A study that examined the effect of GC genetic variability in 913 infants on 25(OH)D concentrations and the response to supplementation demonstrated that vitamin-D-binding protein (DBP) polymorphisms affected the efficacy of vitamin D supplementation and vitamin D status [22]. In addition, similar studies highlighted the role of GC genotypic variation in determining the efficacy of vitamin D supplementation in pregnant women [23].

Physiologically, DBP can play an important role in determining vitamin D status by controlling levels of both total and free vitamin D metabolites. DBP is extremely polymorphic, with variants exhibiting differences in biological function and being associated with several pathophysiological conditions, including susceptibility to viral infections such as hepatitis C [24,25,26]. A study by Batur and Hekim (2019) evaluated the link between polymorphisms of the DBP-encoding gene *GC* at specific loci and COVID-19 positivity/death among several populations across 10 countries. The findings revealed a protective effect for the TT genotype at the rs7041 locus and a susceptibility risk effect for the GT genotype (*p* < 0.05) at the same locus among all populations. The results suggested an important role for the genetic variants of *GC* in explaining disparities in the prevalence of COVID-19 infection and its mortality rates in the context of vitamin D metabolism [27].

Several cytochrome P450 enzymes are involved in vitamin D metabolism. CYP2R1 is one of those enzymes that play a key role in vitamin D hydroxylation [28]. Several researchers have evaluated the relation between genetic variants of CYP2R1 and vitamin D status among populations and have concluded that a robust correlation existed between specific polymorphisms on SNPs (rs10766197 and rs10741657) and risk of vitamin D deficiency [29,30]. Interestingly, knockout experiments in mice showed that CYP2R1 activity on vitamin D hydroxylation could be compensated for by another unidentified enzyme [31].

One study of the Arabian and South Asian populations in Kuwait showed that CYP2R1 SNPs (rs10500804 and rs12794714) were significantly correlated with serum 25(OH)D levels among the Arabian group but not among the South Asians [32]. That finding is in agreement with findings from this study because the population in our investigation consisted predominantly of South Asians, not Arabs (290 individuals from South Asia, 40 Arabs, and 23 from other ethnicities). Further research on the ethnicities of the UAE population with a good sample size is thus needed to yield a broad picture about the implications of genetic determinants of CYP2R1 on vitamin D status and before generalizing findings to all races and ethnicities in the UAE population.

To the best of our knowledge, this study is the first to evaluate the contribution of genetic variants of vitamin D metabolism to COVID-19 disease severity in the context of 25(OH)D levels among a cohort of patients who tested positive for SARS-CoV-2 in the UAE. Our findings could pave the way for future investigations aiming to examine the potential role of variants of vitamin-D-related genes in altering not only COVID-19 disease outcome and transmission in this population but also response to vaccination on the basis of vitamin D status and immune responses. Results could be projected to guide future innovative and unique modeling with other viral respiratory diseases by using a personalized genotype-based approach that could be particularly valuable for multiethnic populations.

Despite the strengths of our study, some limitations should be acknowledged. The association between the genetic polymorphism of the genes studied and mortality was not investigated because of the few deaths in the study. A larger sample size, which affords the opportunity to examine death as an endpoint, could offer more insights into the relation between the genetic determinants of vitamin D status and the death arising from SARS-CoV-2 infection. Moreover, some socioeconomic data were not available to explore the effect of UVB exposure, clothing habits, and dietary vitamin D intake on the contribution of polymorphisms to 25(OH)D concentrations and course of COVID-19. The vitamin D status of participants could have been affected by the use of supplements, but that information was not recorded. In addition, the analytical methods for measuring vitamin D in this study could affect the reading, and therefore, it is suggested that they should be evaluated using LC-MS/MS [33].

## 5. Conclusions

The findings described here showed that variability in the genetic determinants of vitamin D bioavailability and metabolism could play a role in modulating COVID-19 outcome among affected people in the UAE. These preliminary results could pave the way for future investigations about the role of vitamin D status and supplementation in conjunction with genotypic profile in reducing the severity of COVID-19 and risk of infection. The implications of our research could also be informative for improving treatment options by identifying individuals at risk of developing more severe outcomes after infection on the basis of a personalized genetic profile, which could complement the vaccination and vitamin D supplementation approach for improving assessment of treatment. Larger studies and randomized clinical trials are warranted to fully decipher the link between vitamin D homeostasis and genetic determinants and COVID-19 severity among the multiethnic UAE population.

## Figures and Tables

**Table 1:** Demographics of study participants.

Variable	Noncritical, *n* = 453 (%)	Critical, *n* = 193 (%)	*p*
Age			<0.001
<33	155 (34.2)	12 (6.2)	
34–43	138 (30.5)	31 (16.1)	
44–55	92 (20.3)	60 (31.1)	
>56	68 (15.0)	90 (46.6)	
Sex			0.217
Male	305 (77.2)	70 (83.3)	
Female	90 (22.8)	14 (16.7)	
Body mass index (kg/m^2^ of body surface area)			0.001
<18.50	9 (2.3)	1 (0.5)	
18.51–24.90	115 (29.4)	38 (19.7)	
24.91–29.99	168 (43.0)	77 (39.9)	
>30.00	99 (25.3)	77 (39.9)	
Country of origin			0.004
Middle Eastern	40 (30.9)	80 (41.5)	
Asian	290 (64.0)	103 (53.4)	
African	18 (4.0)	3 (1.6)	
European	2 (0.4)	5 (2.6)	
American	3 (0.7)	2 (1.0)	
History of comorbid condition			<0.001
No	263 (65.1)	75 (40.3)	
Yes	141 (34.9)	111 (59.7)	
Diabetes			<0.001
No	279 (75.4)	78 (47.3)	
Yes	91 (24.6)	87 (52.7)	
Immunosuppressive condition			0.20
No	250 (93.6)	117 (90.0)	
Yes	17 (6.4)	13 (10.0)	
Liver disease			0.01
No	323 (99.4)	128 (96.2)	
Yes	2 (0.6)	5 (3.8)	
Metabolic disease			<0.001
No	318 (95.8)	112 (83.0)	
Yes	14 (4.2)	23 (17.0)	
Neurological disorder			0.03
No	320 (98.8)	126 (95.5)	
Yes	4 (1.2)	6 (4.5)	
Renal disease			<0.001
No	312 (94.8)	111 (78.7)	
Yes	17 (5.2)	30 (21.3)	
Cardiac condition			<0.001
No	312 (91.5)	111 (76.0)	
Yes	29 (8.5)	35 (24.0)	
Chronic lung condition			<0.001
No	318 (97.2)	113 (86.3)	
Yes	9 (2.8)	18 (13.7)	

**Table 2:** Association of 25(OH)D status with risk of critical COVID-19 disease.

25(OH)D Status (ng/mL)	Noncritical, *n* = 322 (%)	Critical, *n* = 155 (%)	*p*	Unadjusted OR (95% CI)	*p*	Adjusted OR (95% CI)	*p*
Normal (>20)	90 (28.0)	56 (36.1)	0.02	1.00		1.00	
Deficient (<20)	142 (44.1)	47 (30.3)		0.54 (0.35–0.89)	0.008	1.28 (0.62–2.65)	0.49
Severely deficient (<12)	90 (28.0)	52 (33.5)		0.95 (0.59–1.51)	0.93	4.37 (2.06–9.29)	<0.001

**Table 3:** Association of analyzed genotypes with risk of critical COVID-19 disease.

Gene	SNP	Genotype	Noncritical, *n* = 453 (%)	Critical, *n* = 193 (%)	Genetic Model	Unadjusted OR (95% CI)	*p*	Adjusted OR (95% CI)	*p*
***GC***	rs59241277	AA AG GG	363 (80.1) 86 (19.0) 4 (0.9)	174 (90.2) 18 (9.3) 1 (0.5)	AA vs. AG + GG	0.47 (0.28–0.77)	0.003	0.43 (0.24–0.77)	**0.005**
	rs113574864	CC CT TT	350 (77.3) 96 (21.2) 7 (1.5)	168 (87.0) 23 (11.9) 2 (1.1)	CC vs. CT + TT	0.47 (0.29–078)	0.003	0.43 (0.24–0.78)	**0.005**
	rs182901986	GG GA AA	354 (78.1) 93 (20.5) 6 (1.3)	171 (88.6) 20 (10.4) 2 (1.0)	GG vs. GA + AA	0.50 (0.31–0.80)	0.003	0.49 (0.29–0.84)	**0.01**
	rs60349934	TT TC CC	359 (79.2) 91 (20.1) 3 (0.7)	170 (88.1) 22 (11.4) 1 (0.5)	TT vs. TC + CC	0.55 (0.34–0.87)	0.011	0.50 (0.28–0.86)	**0.01**
	rs113876500	GG GT TT	350 (77.3) 96 (21.2) 7 (1.5)	168 (87.1) 23 (11.9) 2 (1.0)	GG vs. GT + TT	0.54 (0.35–0.84)	0.006	0.53 (0.31–0.88)	**0.02**
***NADSYN1***	rs4944076	AA AG GG	148 (32.7) 198 (43.7) 107 (23.6)	78 (40.4) 93 (48.2) 22 (11.4)	AA vs. AG + GG	0.66 (0.51–0.84)	0.001	0.66 (0.49–0.90)	**0.008**
	rs4944997	GG GA AA	144 (31.8) 199 (43.9) 110 (24.3)	73 (37.8) 95 (49.2) 25 (13.0)	GG vs. GA + AA	0.69 (0.54–0.89)	0.004	0.69 (0.51–0.93)	**0.02**
	rs4944998	GG GC CC	144 (31.8) 200 (44.2) 109 (24.1)	73 (37.8) 95 (49.2) 25 (13.0)	GG vs. GC + CC	0.70 (0.55–0.89)	0.004	0.70 (0.51–0.94)	**0.02**
	rs4944979	GG GT TT	146 (32.2) 197 (43.5) 110 (24.3)	74 (38.3) 94 (48.7) 25 (13.0)	GG vs. GT + TT	0.69 (0.54–0.89)	0.004	0.70 (0.52–0.94)	**0.02**
	rs10898210	AA AG GG	157 (34.7) 196 (45.2) 100 (22.1)	85 (44.0) 87 (45.1) 21 (10.9)	AA vs. AG + GG	0.64 (0.50–0.82)	0.0005	0.67 (0.50–0.90)	**0.009**
***VDR***	rs11574018	TT TC CC	438 (96.7) 15 (3.3) 0 (0.0)	192 (99.5) 1 (0.5) 0 (0.0)	TT vs. TC + CC	0.15 (0.02–1.17)	0.051	0.10 (0.01–0.86)	**0.04**
	rs11574024	GG GT TT	440 (97.1) 13 (2.9) 0 (0.0)	190 (98.4) 3 (1.6) 0 (0.0)	GG vs. GT + TT	0.53 (0.15–1.89)	0.328	0.20 (0.04–0.91)	**0.04**
	rs116886958	CC CA AA	428 (94.5) 25 (5.5) 0 (0.0)	187 (96.9) 6 (3.1) 0 (0.0)	CC vs. CA + AA	0.55 (0.22–1.36)	0.195	0.37 (0.13–1.04)	0.06
	rs10875694	TT TA AA	359 (79.2) 90 (19.9) 4 (0.9)	158 (81.9) 32 (16.6) 3 (1.6)	TT vs. TA + AA	0.90 (0.60–1.33)	0.602	0.63 (0.13–1.02)	0.06
	rs2239181	AA AC CC	429 (94.7) 24 (5.3) 0 (0.0)	176 (91.2) 15 (7.8) 2 (1.0)	AA + AC vs. CC	1.00 (0.71–1.41)	0.996	1.44 (0.95–2.18)	0.08
***CYP2R1***	rs11023373	GG GC CC	426 (94.0) 23 (5.1) 4 (0.9)	188 (97.4) 5 (2.6) 0 (0.0)	GG vs. GC + CC	0.37 (0.14–0.95)	0.034	0.39 (0.13–1.15)	0.09
	rs11023374	TT TC CC	284 (62.7) 147 (32.5) 22 (4.9)	130 (67.4) 55 (28.5) 8 (4.1)	TT vs. TC + CC	0.84 (0.62–1.14)	0.271	0.73 (0.51–1.05)	0.09
	rs10500804	TT TG GG	165 (36.4) 209 (46.1) 79 (17.4)	78 (40.4) 83 (43.0) 32 (16.6)	TT vs. TG + GG	0.90 (0.70–1.15)	0.415	0.83 (0.61–1.11)	0.22
	rs1993116	GG GA AA	192 (42.4) 205 (45.3) 56 (12.4)	75 (38.9) 91 (47.2) 27 (14.0)	GG + GA vs. AA	1.11 (0.87–1.43)	0.377	1.24 (0.92–1.69)	0.15
	rs7935792	AA AC CC	362 (79.9) 84 (18.5) 7 (1.5)	157 (81.3) 33 (17.1) 3 (1.6)	AA vs. AC + CC	0.92 (0.62–1.37)	0.703	1.00 (0.61–1.65)	0.99

An additive (dominant) genetic model was applied for the genetic model where the homozygous genotype of the more frequent allele was compared with the heterozygous genotype and homozygous genotype of the less frequent allele. Adjusted for age, sex, population stratification; OR = odds ratio; SNP = single-nucleotide polymorphism. Data are presented as frequencies (%) and OR (95% CI); *p* < 0.05 considered significant (shown in boldface).

## Data Availability

Data are available on request from the first and corresponding authors.

## Supplementary Materials

The following are available online at https://www.mdpi.com/article/10.3390/nu13113680/s1, Table S1: Unadjusted and adjusted (corrected for age, gender and 10 PCAs for population stratification) values of all the SNPs located in gene *GC* (Chr. 4: 72607410-72671237; 168 SNPs), *NADSYN1* (Chr. 11: 71164249-71212862; 230 SNPs), *VDR* (Chr. 12: 48235320-48298777; 154 SNPs) and *CYP2R1* (Chr. 11: 14898986-14913777; 9 SNPs). OR=Odds Ratio; P = *p*-value, Table S2: Genotyping count of the total SNPs located in gene *GC* (Chr. 4: 72607410-72671237; 168 SNPs), *NADSYN1* (Chr. 11: 71164249-71212862; 230 SNPs), *VDR* (Chr. 12: 48235320-48298777; 154 SNPs) and *CYP2R1* (Chr. 11: 14898986-14913777; 9 SNPs).Table S3: Haplotype block composed by the SNPs located in gene *GC* (Chr. 4: 72607410-72671237; 168 SNPs), *NADSYN1* (Chr. 11: 71164249-71212862; 230 SNPs), *VDR* (Chr. 12: 48235320-48298777; 154 SNPs) and *CYP2R1* (Chr. 11: 14898986-14913777; 9 SNPs).
